# Rectovaginal fistula as a complication of rectal injury during vaginal reconstructive surgery: A case report

**DOI:** 10.1016/j.ijscr.2024.109856

**Published:** 2024-06-06

**Authors:** Fernandi Moegni, Anggrainy Dwifitriana Kouwagam, Surahman Hakim, Tyas Priyatini, Alfa Putri Meutia, Budi Iman Santoso

**Affiliations:** Division of Urogynecology and Reconstruction, Department of Obstetrics and Gynecology, Faculty of Medicine, Universitas Indonesia/Dr. Cipto Mangunkusumo National General Hospital, Indonesia

**Keywords:** Rectovaginal fistula, Vaginal agenesis, Vaginal reconstructive surgery

## Abstract

**Introduction and importance:**

Rectovaginal fistula is a complication that may occur due to rectal injury during vaginal reconstructive surgery. To prevent these complications, the recognition of the injury is an important factor so that primary repair can be done. The primary repair can reduce the risk of complications such as fistula formation, and also reduce the physical and psychological impact on the patient.

**Case presentation:**

A 33-year-old woman, came with a chief complaint of fecal leakage from the vagina and abdominal pain three months before admission with a history of vaginal reconstructive surgery due to vaginal agenesis. Eleven years after the reconstruction, the patient was diagnosed with recurrent obstruction caused by vaginal synechia. During the surgery of synechia release, rectum injury occurred. Even though primary closure repair was done at that time, several months later there was a complication of rectovaginal fistule formation in the form of fecal leakage from the vagina. The corrective surgery is performed in collaboration with a surgical gastroenterologist.

**Clinical discussion:**

Iatrogenic rectal injury may occur during gynecological surgery. A fistula that occurs after the reconstruction of vaginal agenesis is a high-type rectovaginal fistula, making the repairs more complex. Collaboration surgery between surgical gastroenterologist and gynecologist may be an option in such cases.

**Conclusion:**

Rectovaginal fistula is a rare but serious complication of vaginal reconstructive surgery. Early recognition, immediate management, and postoperative follow-up are essential in cases of rectal injury during vaginal reconstructive surgery.

## Highlights

• Rectovaginal fistula is a complication that may occur due to rectal injury during vaginal reconstructive surgery.
• The primary repair can reduce the risk of complication such as fistula formation, and reduce the physical and psychological impact on the patient.
• Early recognition and management with postoperative follow-up are essential in cases of rectal injury during vaginal reconstructive surgery.

## 1 Introduction and importance

Abnormalities of the female reproductive tract exhibit varying prevalence in different studies. According to a systematic review by Savarelos et al., the prevalence of female reproductive tract disorders is estimated at 7 % with one of the most common abnormalities being vaginal agenesis [1]. The definitive treatment for vaginal agenesis is surgical reconstruction of a neo vagina, often performed using the McIndoe technique. This procedure carries a risk of injuring the rectum [2,3]. If the rectal injury is known during reconstructive surgery, immediate closure repair can be performed. However, if the repair is unsuccessful, a rectovaginal fistula may develop. Given its impact on the patient's physical and psychological well-being, this condition must be treated appropriately [4].

We present a case of rectovaginal fistula as a complication of rectal injury during vaginal reconstructive surgery. This study has been reported in accordance with the SCARE criteria [5].

## 2 Patient information

A 33-year-old married woman came with complaints of abdominal pain for three months and feces coming out of the vagina. Eleven years before admission, the patient was diagnosed with vaginal agenesis and neovaginal surgery was performed. One year before admission, the patient began experiencing another cyclic abdominal pain along with blocked menstruation. She was subsequently diagnosed with cervicovaginal synechia and underwent a release surgery utilizing a double approach technique: laparotomy hysterotomy, release of tubal adhesions, right cystectomy, and implantation of a mold amnion graft via a vaginal approach. During the intraoperative, a rectal injury, identified as a perforation was diagnosed (located 4 cm behind the introitus with a diameter of 1 cm) and immediate closure repair was performed. However, after the patient has been hospitalized for 7 days when the amnion graft mold was released, she complained of feces coming out of the vagina indicating failure of the closure. She was sent home with an intrauterine 24fr silicone catheter inserted to prevent further vaginal obstruction due to the “dirty” fecal-contaminated vaginal situation. The silicone catheter was changed monthly until the third month when it spontaneously came off, resulting in a reobstructed vaginal canal.

## 3 Clinical finding

**Fig. 1:**
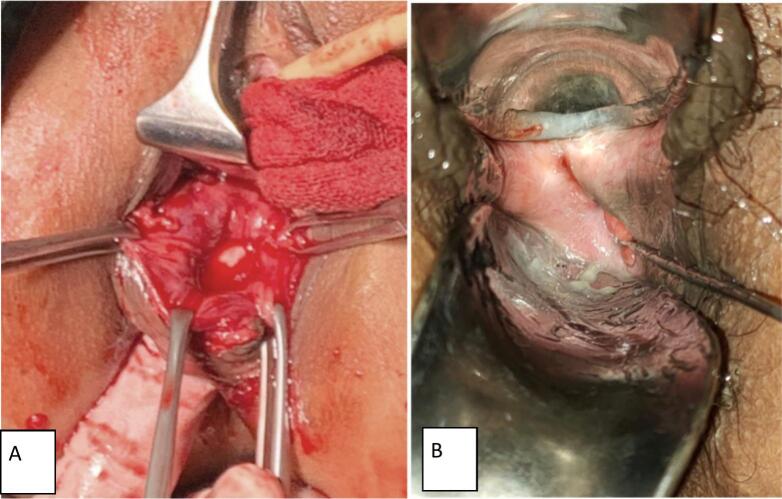
(A) Defect in vagina mucosa during cervical synechia surgery. (B) Defect in vaginal mucosa when cervicovaginal fistula occurred.

There was a palpated abdominal mass, sized 8 × 5 × 6 cm with pain in palpation. Gynecology speculum examination revealed shortened vagina with a total vaginal length was 4 cm. A defect in posterior vaginal mucosa was found at 5 o'clock, connected to the rectum, located 2 cm above the hymen with a diameter of 7 mm (Fig. 1).

From rectovaginal touche, we found obstruction of vaginal canal palpated at 4 cm proximal from anal canal, portio was not palpable, enlarged uterus was palpable with size 8 × 5 × 6 cm. The defect was felt at the posterior vaginal mucosa connected to rectum, a distance 2 cm proximal from the anal canal, with 7 mm diameter.

## 4 Diagnostic assessment

**Fig. 2:**
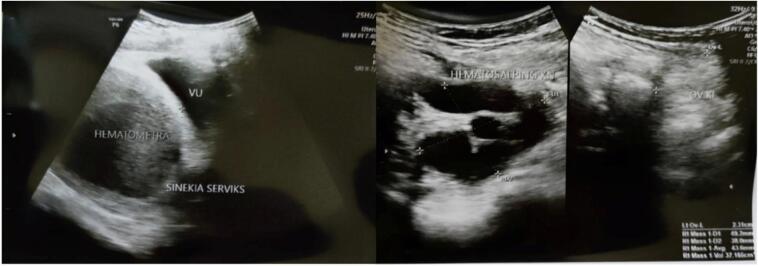
Hematometra, hematosalphinx and cervical synechia.

From urogynecology ultrasound, there were cervical synechia, hematometra and right hematosalphinx (Fig. 2).

## 5 Intervention

**Fig. 3:**
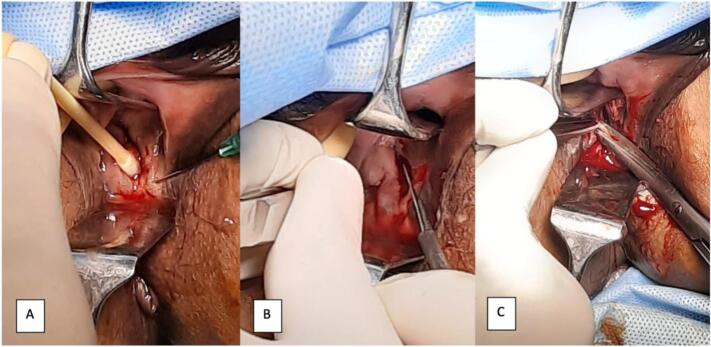
(A) Hydrodissection along the edge of the fistula, (B) incision of the fistula edge undermining vaginal mucosa, (C) closure layer by layer.

**Fig. 4:**
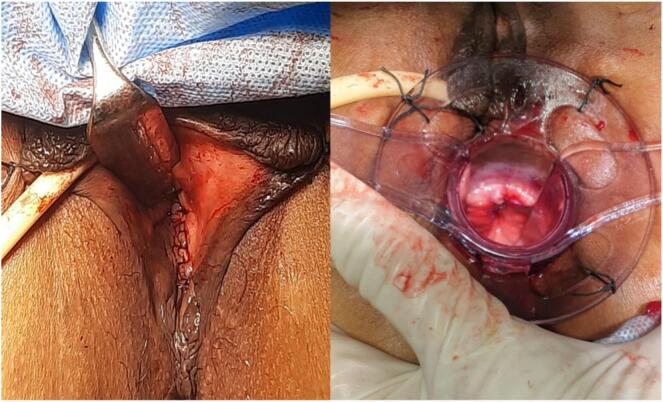
Final view after repair.

Patient was diagnosed with hematometra, right hematosalpinx, cervicovaginal synechia, and rectovaginal fistulae. After counseled about management options, patient was agreed to perform total abdominal hysterectomy, right salphingectomy and rectovaginal fistula repair through a joint operation involving an experienced urogynecologist and surgical gastroenterologist. The fistula repair involved hydrodissection along the edge of the fistula, followed by incision of the fistula edge to undermine the vaginal mucosa. The procedure concluded with closure of the rectal mucosa, followed by closure of the vaginal mucosa (Fig. 3, Fig. 4).

## 6 Follow up and outcome

Patient was discharged three days after operation, no fecal matter found in neovagina. Three months follow up revealed a rather satisfying outcome with wexner score of 0.

## 7 Discussion

Although a rare condition, iatrogenic rectal injury during gynecological surgery can lead to serious complications in the future. Recognizing rectal injury at the time of surgery offers a better prognosis. Therefore, a digital rectal examination can be performed to help diagnose the rectal injury. Other methods include bubbling air into the rectum while filling the pelvis with irrigation fluid and inserting an intrarectal device such as a balloon or bougie for better visualization of the anterior wall [6].

Systematic diverting colostomy was previously recommended for managing rectal injuries, but several studies have demonstrated the effectiveness of primary repair. Primary repair statistically reduces the incidence of complications such as an intra-abdominal abscess or wound dehiscence. In the study of Jo et al., a two-layer closure was adequate to treat rectal injuries. Factors including transfusion requirements, the patient's physical condition, antibiotic use, type and location of injury, and degree of colonic damage can be considered when deciding between diversion or primary repair in cases of rectal injury. Colonic tissue that cannot be repaired should be treated by diverting the injured colon [6].

Rectal injury repair can be performed by a surgeon or urogynecologist. Jo et al. noted that the results of surgery by surgeons and gynecologists did not differ significantly. Surgeons may be required in cases of sepsis complications due to extensive damage. Injuries less than 3 cm can still be repaired by a gynecologist who is capable of delicate suturing without increasing the risk of postoperative complications [4,6].

A rectovaginal fistula is an abnormal communication between the rectum and the neovagina after vaginoplasty in cases of vaginal agenesis. This condition can lead to uncontrollable passage of fecal material into the neovagina, causing morbidities such as local infection, urinary tract infection, and psychological distress for the patient [4].

The McIndoe method is a widely used method to treat cases of vaginal agenesis. If a fistula occurs after the McIndoe method, it is classified as a high-type fistula. Dissection of the apex vaginal pocket may pose challenges in visualizing and tactically sensing the tissue. Excessive efforts to reach the rudder or an unformed cervix can result in rectal injury, potentially leading to a fistula. Symptoms include fecal odor in the neovaginal fluid and the presence of fecal matter in the neovagina. Once symptoms are present, a fistula has typically formed [4].

There are several methods for treating rectovaginal fistulas based on the anatomic location. These methods include the Martius flap-labia fat and bulbocavernosus muscle passed subcutaneously to cover the damage, gracilis muscle interposition or rectus muscle interposition, omental pedicle graft, and rectus abdominis flap graft. Following the formation of a rectovaginal fistula, the patient should undergo a diverting colostomy to prevent continuous fecal leakage into the neovagina, which may hinder healing and lead to infection [4].

Challenges encountered during fistula repair include the thinness of the posterior neovaginal wall due to the absence of normal vaginal mucosa and submucosal connective tissue. In addition, skin graft contractures also increase the difficulty of the operation. The graft flaps that are formed require good vascularity to resettle. Gracilis muscle interposition is a suitable method as it provides sufficient vascularization for grafting the posterior neovaginal wall [4].

Corrective surgery should be performed at least 8–12 weeks after fecal diversion, once neovaginal tissue has healed and skin grafting has matured. An anovaginal examination can detect the presence of a rectovaginal fistula. If persistent depression is suspected, an examination can be performed using a vaginal tampon. Methylene blue is then injected into the rectum with the anal orifice closed for 15–20 min. If the tampon is stained, a fistula is suspected [7].

Fistulas less than 2.5 cm, generally due to trauma or infection, fall into the simple category. A recommended technique involves a straight suture with vaginal perineal access or transanal access, followed by a mucosal advancement flap. Lowery et al. reported an 88 % success rate using this technique in the primary repair of simple fistulas. The more often the fistula is repaired, the success rate decreases. This is associated with the presence of recurrent inflammation, tension, hematoma, or underlying diseases. In this study, it was advisable to wait 3–6 months before performing a second repair to reduce the acute phase of infection and inflammation [7].

In our case, the rectal injury was a complex type with a diameter of more than 1 cm. Based on the location, most likely the rectal injury that occurred is a high type. The patient had undergone reconstruction of vaginal agenesis with the McIndoe method which might be the cause of prone to rectal injury. Thus, the high location and the diameter make repairs more complex. Collaboration surgery between digestive surgery and a gynecologist may be an option in such cases. This case also becomes more complex because the patient has undergone several surgeries like ovarian cyst surgery, vaginal reconstruction, and synechia release so larger fibrotic tissue has formed. The operation becomes more difficult so that damage to surrounding organs cannot be avoided such as rectal injury. Follow-up seven days post-op should be done, especially with the methylene blue method to determine the formation of a fistula.

## 8 Conclusion

A rectovaginal fistula is a rare but serious complication of vaginal reconstructive surgery. Early recognition, immediate management, and postoperative follow-up are essential in cases of rectal injury during vaginal reconstructive surgery.

## Provenance and peer review

Not commissioned, externally peer-reviewed.

## Ethical approval

This study was exempt from ethical approval by our institution because all actions, examination, and procedure were done according to hospital's standard of procedure and policy.

## Funding

The study was fully funded by the authors.

## Research registration number

Not applicable.

## Consent

Written informed consent was obtained from the patient for publication and any accompanying images. A copy of the written consent is available for review by the Editor-in-Chief of this journal on request.

## Conflict of interest statement

The authors report no declarations of interest.

## References

[1] Saravelos S.H., Cocksedge K.A., Li T.C. (2008). Prevalence and diagnosis of congenital uterine anomalies in women with reproductive failure: a critical appraisal. Hum. Reprod. Update.

[2] Kölle A., Taran F.A., Rall K., Schöller D., Wallwiener D., Brucker S.Y. (2019 Oct). Neovagina creation methods and their potential impact on subsequent uterus transplantation: a review. BJOG.

[3] Setchell M.E., Hudson C.N. (2013).

[4] Rao R.S., Rao J.S., Deora H., Gupta R. (2015). Recto-neovaginal fistula following vagino-plasty for vaginal agenesis: our experience and management guidelines. Sch. J. App. Med. Sci..

[5] Sohrabi C., Mathew G., Maria N., Kerwan A., Franchi T., Agha R.A. (2023). The SCARE 2023 guideline: updating consensus Surgical CAse REport (SCARE) guidelines. Int. J. Surg. Lond. Engl..

[6] Jo E.J., Lee Y., Kim T., Choi C.H., Lee J., Bae D. (2012). Management and outcome of rectal injury during gynecologic laparoscopic surgery. JMIG.

[7] Danzi M., Massimilliano F., Stefano R., Mario P., Bruno A., Luciano G. (2013). Surgical mistake causing an high recto-vaginal fistula. A case report with combined surgical and endoscopic approach therapeutic considerations. BMS Surg..

